# Equine Models of Temporomandibular Joint Osteoarthritis: A Review of Feasibility, Biomarkers, and Molecular Signaling

**DOI:** 10.3390/biomedicines12030542

**Published:** 2024-02-28

**Authors:** Tomasz Jasiński, Bernard Turek, Michał Kaczorowski, Walter Brehm, Katarzyna Skierbiszewska, Joanna Bonecka, Małgorzata Domino

**Affiliations:** 1 Department of Large Animal Diseases and Clinic, Institute of Veterinary Medicine, Warsaw University of Life Sciences (WULS-SGGW), 02-787 Warsaw, Poland; tomasz_jasinski@sggw.edu.pl (T.J.); katarzyna_skierbiszewska@sggw.edu.pl (K.S.); 2 Private Equine Practice, 05-825 Grodzisk Mazowiecki, Poland; mskaczorowski@gmail.com; 3 Department for Horses, Veterinary Teaching Hospital, University of Leipzig, 04103 Leipzig, Germany; brehm@vetmed.uni-leipzig.de; 4 Department of Small Animal Diseases and Clinic, Institute of Veterinary Medicine, Warsaw University of Life Sciences (WULS-SGGW), 02-787 Warsaw, Poland; joanna_bonecka@sggw.edu.pl

**Keywords:** temporomandibular joint, osteoarthritis, cartilage degeneration, mechanical loading, remodeling, regulation, targeted treatment, horse, human

## Abstract

Osteoarthritis (OA) of the temporomandibular joint (TMJ) occurs spontaneously in humans and various animal species, including horses. In humans, obtaining tissue samples is challenging and clinical symptoms appear late in the disease progression. Therefore, genetically modified, induced, and naturally occurring animal models play a crucial role in understanding the pathogenesis and evaluating potential therapeutic interventions for TMJ OA. Among the naturally occurring models, the equine TMJ OA model is characterized by slow, age-related progression, a wide range of clinical examinations, and imaging modalities that can be performed on horses, as well as easy tissue and synovial fluid collection. The morphological and functional similarities of TMJ structures in both species make the equine model of TMJ OA an excellent opportunity to track disease progression and response to treatment. However, much work remains to be carried out to determine the utility of human TMJ OA biomarkers in horses. Among the main TMJ OA biomarkers, IL-1, IL-6, TGF-β, TNF-α, and PGE_2_ have been recently investigated in the equine model. However, the majority of biomarkers for cartilage degradation, chondrocyte hypertrophy, angiogenesis, and TMJ overload—as well as any of the main signaling pathways—have not been studied so far. Therefore, it would be advisable to focus further research on equine specimens, considering both mediators and signaling.

## 1. Introduction

Osteoarthritis (OA) stands out as the most prevalent degenerative disease affecting the temporomandibular joint (TMJ), manifesting spontaneously in humans [1] and various animal species, such as mice [2,3], guinea pigs [4], and horses [5,6,7,8,9,10]. TMJ OA is characterized as a chronic disease marked by degenerative alterations in cartilage, accompanied by reparative processes in the surrounding tissues [1,11,12,13,14]. As the disease causes severe pain [12], joint swelling, and joint stiffness [1], limitation of mandibular motion [13] and subsequently a decreased quality of life [14] may be expected. The etiology of TMJ OA is complex and multifactorial, generally attributed to factors such as mechanical overloading, abnormal occlusion, trauma, and stress [1,11,15]. However, the specific causes of impaired cartilage and subchondral bone in the TMJ remain unclear [14,16], necessitating further research.

In the research, obtaining tissue samples from humans with TMJ OA poses challenges, and clinical symptoms often manifest late in the disease progression [14]. Consequently, animal models of TMJ OA play a pivotal role in comprehending the pathogenesis and assessing potential therapeutic interventions [17,18]. Given the morphological and functional differences in the TMJ across species, it is acknowledged that no single animal species can serve as a comprehensive model for all aspects of human TMJ pathophysiology [19]. Animal models of TMJ OA are therefore categorized into five groups: genetically modified, induced (surgically, chemically, or non-invasively), and naturally occurring models, depending on whether animals undergo intervention or not [14,20,21]. Genetically modified mice offer valuable insights into the molecular basis of TMJ OA by allowing the direct observation of individual gene influences [12,16]. However, human TMJ OA pathogenesis involves multiple genes [22] and genetically modified mice may not fully replicate the intricate multi-gene interactions [14]. Surgical induction models employing mice, rats, rabbits, pigs, and sheep are common [14], with rabbits preferred due to favorable outcomes [23,24]. Chemically induced models involving mice, rats, rabbits [14], and horses [25] are also widely utilized, with rats preferred due to low costs and ease of management [26,27]. Rats are frequently employed in non-invasive models using mechanical loading due to their tolerance to such devices [28,29]. However, induced models typically operate on one TMJ [14,23,24,25,26,27,28,29], overlooking its bilateral nature [30]. Non-invasive models overlook this limitation but face challenges due to differences in occlusion, TMJ movements, and TMJ structure between rodents and humans [1,31]. Therefore, larger animals like pigs, sheep, and horses, with greater medio-lateral TMJ mobility, are increasingly recommended [12].

Amongst large animal models, only horses exhibit the naturally occurring slow progression of TMJ OA [5,6,7,8,9,10], closely resembling the disease progression in humans [14,32]. Moreover, horses, similarly to humans [33], experience intra-articular disc mineralization, fractures involving TMJ, and septic arthritis [6,7,8,34], offering a promising platform for translational research. The equine naturally occurring model of TMJ OA offers significant advantages including the elimination of the need for invasive procedures [14], minimization of the potential side effects [14], and providing a means to study the pathophysiology, biological markers, and therapeutic agents [33,35]. Consequently, the improvement of the equine naturally occurring model addresses the need for more responsive outcome measures for both symptom- and structure-modifying agents for human TMJ by developing and qualifying biomarkers to enable the development of disease-modifying therapeutic strategies [12,16]. On the other hand, the utility of the equine model of TMJ OA is evident in tracking the course of the disease in veterinary medicine, particularly in equine sports medicine. Diagnosis and treatment of equine TMJ OA is a major clinical challenge as TMJ OA may significantly impair the performance of equine athletes [36,37]. Despite the development of several equine models for peripheral joint OA [35], TMJ OA still presents deficiencies with inconsistency in the level of disease between animals [6] and challenges in establishing biomarkers and signaling pathways for tracking TMJ OA [5].

This review sought to provide a general comparison of TMJ OA in humans and horses. Then, this review aims to focus on summarizing the currently investigated markers and signaling pathways in TMJ OA, discussing the gaps in their state of the art within the context of the equine model of TMJ OA.

## 2. TMJ OA in Humans and Horses

The equine OA models are anatomically and pathophysiologically similar to humans, particularly in cartilage thickness, spontaneous OA occurrence, age-related progression, pain-related clinical symptoms, radiographic signs, imaging modalities used, risk factors for OA development, and similar intra-articular OA mediation [33]. In horses, the OA model of the TMJ is still poorly understood [38,39,40,41], in contrast to equine OA models of the peripheral joints. In recent well-published research, significant similarities between the human knee joint OA and the equine metacarpophalangeal (MCP) joint OA [21,32,35] and stifle joint OA [32,42,43] have been evidenced. Additionally, a racehorse model was established as a specific joint-loading model used to measure the impact of effort on knee OA development, since microstructural changes in articular cartilage due to overloading of the equine joint were evidenced [44,45]. As the OA may progress differently in different joints [46], more research is still needed in equine TMJ OA, especially in the area of markers and signaling pathways.

OA occurs spontaneously in humans and equines in both peripheral joints and the TMJ [1,5,6,7,8,9,12,15]. The occurrence of slowly progressing equine OA closely resembles the natural progression of human primary OA [32]. Moreover, spontaneous OA is a common clinical problem in both humans [12,13,33,47,48,49] and horses [7,8,37,38], also in the TMJ. Focusing on TMJ OA in humans, one can observe that clinical symptoms of TMJ OA have been reported in 40–75% of adults [13], 67% of adults with coexisting OA in other joints [48], and in more than 70% of older people [49]. In horses, radiographic signs of TMJ OA have been found in over 30% of examined horses [6]. Moreover, the frequency of TMJ OA increases with age in both humans [1,50,51] and horses [6,8,10]. Clinically asymptomatic horses accumulate changes in the TMJs with age [10], similar to those seen in the TMJs of other species, including humans [32]. Like humans, horses experience age-related degeneration in the form of intra-articular disc mineralization [6,7] and changes in intra-articular proinflammatory cytokine profiles [5].

The main manifestations of TMJ OA include damage and degeneration of the articular cartilage, accompanied by remodeling of the surrounding tissue, beginning with the subchondral bone and progressively involving the synovium and other soft tissues [14]. As a result, alterations may manifest in all articular structures, including the articular cartilage, articular disc, subchondral bone, synovium, joint capsule, ligaments, and periarticular muscles [1,15,17]. As in peripheral joints, the role of synovitis in the pathogenesis of OA has been proven to be similar in humans [52] and horses [53,54]; it can be suspected in the TMJ as well. Moreover, equine articular cartilage is highly comparable to that of humans [32]. In the equine stifle joint, the articular cartilage has been shown to be very similar in thickness and cellular structure, and the biochemical makeup and properties of the cartilage are comparable to the human knee joint [42,43]. Thus, horses have been used to investigate articular cartilage repair and osteochondral defects [21,53,55]. Although the articular surface of the mandibular condyle is covered with fibrous cartilage, consisting of a mass of collagen fibers, instead of hyaline cartilage [56], some similarities may be suspected. Focusing specifically on the TMJ, like humans, naturally occurring degenerative changes in the equine TMJ may impact the compressive stiffness of the intra-articular disc in a region-dependent fashion [57,58]. The composition and mechanical properties of the equine intra-articular disc of the TMJ may deteriorate depending on the horse’s age, the region of the TMJ, and the specific degenerative changes [9]. Continuing the discussion toward the bones, one may observe that horses provide a naturally occurring model to study bone remodeling, which leads to bone cysts and osteophyte formation [6,59].

Among the clinical symptoms of TMJ OA, pain and limited function are the primary reasons for patients to seek treatment. In humans, severe pain, eating difficulties [12], joint swelling, joint stiffness [1], and limited mandibular motion [13] are reported. In the case of horses, which do not report pain themselves, aversion behavior suggestive of pain [8] and problems with horse riding [36,37] are reported by owners or trainers. In clinical examinations, effusion of the affected TMJ and distortion of the masticatory cycle [8,41] are detected. Thus, regardless of the reporting method, similar clinical symptoms are reported in both species, similarly decreasing their quality of life [14,40]. The outcome of clinical TMJ examination is routinely supported by various imaging modalities including conventional radiography, high-resolution ultrasonography, computed tomography (CT), and magnetic resonance imaging (MRI) [14]. Currently, CT and MRI are the most widely used imaging modalities in both humans [1,51,60] and horses [6,61,62], making radiographic signs a key feature for diagnosing TMJ OA. Radiographic signs of the mandibular condyle and zygomatic process of the temporal bone include flattening of the surface, erosive resorption, irregularities of the joint surfaces, subchondral bone sclerosis, osteophyte formation, and cyst-like changes. Similar bone changes can be observed on CT images in both humans [51,63] and horses [6,7,41,59]. Cartilage defects can be visualized using arthroscopy, more often in horses [34,59,64] than in humans [65], and MRI, on the contrary, more often in humans [49,60,66,67,68] than in horses [62,69].

One may highlight the functional similarities between the human and horse TMJs. When considering TMJ load, the horse’s mastication cycle consists of an opening stroke, a closing stroke, and a power stroke [57,70]. The power stroke in horses is unimodal, involving a medio-lateral movement of the mandibles [57]. However, equine TMJs are also capable of latero-ventral movement of the working side during the opening stroke and a marked medio-dorsal movement of the working side during the power stroke [71]. This mastication type is much more similar to humans [72] than the mastication type observed in rodents [1]. The mandibular condyles of rodents extend antero-posteriorly, while in humans, they extend medio-laterally [31]. Thus, the mandibular condyle axis is transversal in humans for tridimensional motions, including opening, deduction, and propulsion, whereas it is sagittal in rodents for propulsion movement [72]. Like humans, the transversal axis of equine mandibular condyles provides tridimensional motions which are provided by the cooperation of the temporal muscle, masseter muscle, medial pterygoid muscle, and lateral pterygoid muscle [61,73]. Such a structure allows for adjustment of the masticatory cycle when compensation in the case of TMJ OA is required; therefore, horses with experimentally induced unilateral TMJ OA do not hesitate to eat and do not show significant pain on TMJ palpation [8,25]. Additionally, the biochemical composition of articular discs in horses [57,58] is similar to that of goats, pigs, bovines, and humans [19]. Moreover, the similarity also extends to the anisotropy of the composition and compressive stiffness of the articular disc. In horses, the orientation of disc anisotropy is similar to that in humans, albeit to a higher degree than observed in the human articular disc [9]. Thus, the regional variations in articular disc composition and compressive stiffness [9] align with the regional distribution of biomechanical stresses and preferred movement directions [57].

These morphological and functional similarities are in line with one fundamental mechanism of OA development, which is an ‘abnormal’ loading on ‘normal’ cartilage [35]. The second one, a ‘normal’ loading on ‘abnormal’ cartilage [35], required further research. It is still not known how the equine TMJ, including the intra-articular disc with ‘normal’ or ‘abnormal’ composition [57,58], withstands ‘normal’ or ‘abnormal’ medio-lateral loading during mastication [9]. Understanding the effect of compressibility on overall TMJ function may, through functional assessment and biomarker evaluation [12,16], shed new light on the pathophysiology of equine TMJ OA and its translation to humans [33]. Such investigations may involve the evaluation of OA biomarkers, which are most effectively obtained directly from the joint [21]. One may note that sampling synovial fluid, articular cartilage, and articular disc in the study of TMJ OA in humans is limited [14,35]. However, these specimens can be effectively investigated using equine OA models [74]. In contrast, the sampling of synovial fluid in the small joints of small animal models is not always feasible [32]. Although biomarkers can be measured from other specimens, such as blood or urine, their levels are influenced by other diseases or metabolic conditions. This limitation is also recognized in human clinical studies [33,49,51,65,66]. Therefore, every effort should be made to establish more and more biomarkers in the equine TMJ OA model.

**Figure 1:**
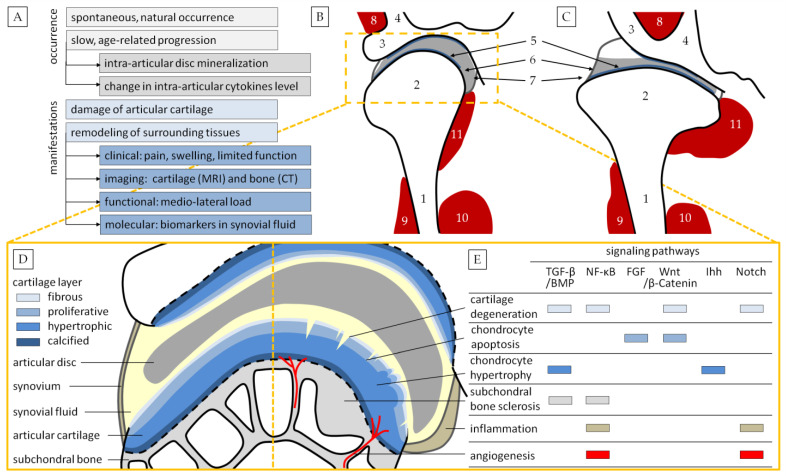
Comparison of temporomandibular joint osteoarthritis (TMJ OA) in humans and horses. A concise summary of occurrence and manifestation (**A**). Schematic representation of the middle plane transverse section of TMJ in humans (**B**) and horses (**C**). 1. Ramus of the mandible; 2. mandibular condyle; 3. zygomatic process of the temporal bone; 4. squamous part of the temporal bone; 5. articular cartilage; 6. articular disc; 7. joint capsule with ligaments; 8. temporal muscle; 9. masseter muscle; 10. medial pterygoid muscle; 11. lateral pterygoid muscle. Schematic representation of normal TMJ (**D**) and TMJ OA (**E**). Abbreviations: TGF-β/BMP, transforming growth factor β/bone morphogenic protein signaling; NF-κB, nuclear factor kappaB signaling; FGF, fibroblast growth factor signaling; Wnt/β-Catenin signaling; Ihh, Indian hedgehog signaling; Notch signaling.

Figure 1A–C summarize the main similarities between human and equine TMJ OA, while Figure 1D,E illustrate the main signaling pathways of TMJ OA initiation and progression, indicating molecules that can be used as TMJ OA biomarkers.

## 3. Biomarkers of TMJ OA

The TMJ function remains in balance with occlusion, thanks to remodeling, which is the essential biological response to TMJ loading [15]. The TMJ exhibits high adaptability to variable load, as its fibrocartilage is highly resistant to shear force [75]. However, when the load exceeds the joint adaptation level, degenerative changes may be initiated. Correlations between occlusal interferences, nonworking-side occlusal contacts, and TMJ OA in adult humans were demonstrated [76], indicating that excessive or prolonged TMJ overload may result in incorrect remodeling [15]. Thus ‘abnormal’ loading on ‘normal’ cartilage may be considered as one of the mechanisms of TMJ OA development [35]. On the other hand, when TMJ adaptability to load is reduced [15], ‘normal’ loading on ‘abnormal’ cartilage [35] may initiate a disruption in the remodeling of the TMJ [17].

‘Abnormal’ cartilage may be produced as a consequence of compromised chondrocyte activity and survival [12], leading to abnormal extracellular matrix (ECM) metabolism [77]. This results in fibrillation, erosion, and cracking in the superficial cartilage layer [14], remodeling of the subchondral bone [15,78], and hastening of the progression of TMJ OA [12]. Chondrocytes mediate the balance of the cartilage matrix [12], and their abnormal anabolic and catabolic activity [77] leads to disruption between ECM synthesis and degradation [12]. In the case of OA, the number of hypertrophic and apoptotic chondrocytes significantly increases [79,80]. Chondrocyte apoptosis results in a decrease in the total number of chondrocytes, creating space for angiogenesis [80]. Through apoptosis, the suppression of autophagy occurs. Autophagy protects chondrocytes against environmental alterations by destroying stored materials and releasing reusable tiny molecules, making it a crucial chondrocyte survival strategy [81]. Chondrocytes with a suppressed survival strategy release apoptotic bodies [80], constituting the origin of cartilage mineralization. This mineral deposition in the ECM [82] and osteoclast activity [15,68] are also stimulated by angiogenesis, so that newly created vessels enter the cartilage and are integrated with osteophytes [82]. Thus, vascular endothelial growth factor (VEGF), crucial for angiogenesis, is considered a key modulator of TMJ OA [82,83]. Hypertrophic chondrocytes induce the degradation of the ECM and calcification of cartilage [80], activating the complement system and the production of cartilage-degrading molecules. Since the cartilage ECM is mainly structured with collagen fibers, mostly type I and type II collagen [84], and proteoglycans [85], the synthesis of matrix metalloproteinases (MMPs, mainly MMP-3, 7, 8, 9, 13, 16, 17, and 19), and other degradative molecules such as a disintegrin and metalloproteinase production with thrombospondin motifs (ADAMTS, mainly ADAMTS-4 and -5) and prostaglandin E_2_ (PGE_2_) initiate collagen and proteoglycan degradation in articular cartilage [12,15,85,86].

Cartilage modeling is often accompanied by bone remodeling related to a decrease in osteoblasts’ activity and amount, as well as an increase in osteoclasts’ activity [87,88]. Osteoblasts participate in the subchondral bone sclerotization so that newly created bone has a high density and volume but low mineralization [89], whereas osteoclasts are involved in bone resorption [90]. Moreover, osteocytes, which are sensitive to joint mechanical loading [91], increase MMP secretion in response to overload, thus promoting the degeneration of the bone matrix and leading to subchondral bone remodeling [92]. On the other hand, the decrease in the sensitivity of chondrocytes to mechanical loading is mediated by the decreased secretion of high mobility group protein B2 (HMGB2) [93] and the activation of hypoxia-induced transcription factor-1 (HIF-1), which promote osteoclastogenesis and VEGF expression [94,95]. VEGF, produced by chondrocytes, stimulates autocrinely the production of MMP-13 and reduces the production of tissue matrix metalloproteinase inhibitor (TIMP-1) [82,94], leading to cartilage degradation [15,68].

These processes occurring in articular cartilage and subchondral bone are accompanied by an inadequate inflammatory response, where immune cells release inflammatory mediators, such as cytokines and chemokines [15,78]. Among them, interleukin 12 (IL-12) [96] and IL-1β [96,97,98,99,100,101,102] seems to be the most important. However, concentrations of IL-2, IL-6, IL-17, IL-18, tumor necrosis factor (TNF-α and TNF-β), and interferon (IFN-γ) were also higher in the synovial fluid from patients with TMJ OA than that of healthy ones [12,96]. IL-1β and TNF-α activate osteoclasts leading to bone resorption [97] and upregulated MMP production in chondrocytes suppressing ECM synthesis, thus contributing to articular and disc cartilage degradation [98]. IL-1β and TNF-α [99], as well as PGE_2_ [103], act in TMJ pain sensation by stimulating nociceptive receptors. IL-1β also stimulates synoviocytes to monocyte chemoattractant protein-1 (MCP-1) production [100], triggering the persistence of inflammation [101]. IL-1β and IL-6 stimulate VEGF transcription in the nucleus and, thus, increase angiogenesis [102]. Due to increased angiogenesis, all these molecules may act not only locally but also by distribution throughout the entire joint [82].

The deregulation of chondrocytes, remodeling of subchondral bone, and activation of immune cells occur as a consequence of a sequence of molecular changes in the TMJ’s tissues. The six main signaling pathways in TMJ OA include transforming growth factor β (TGF-β)/bone morphogenic protein (BMP) signaling, nuclear factor kappaB (NF-κB) signaling, fibroblast growth factor (FGF) signaling, Wnt/β-Catenin signaling, Indian hedgehog (Ihh) signaling, and Notch signaling [12,16]. Table 1 summarizes their members, biological role, activation effects, and role in TMJ OA pathogenesis.

### 3.1. Search Strategies and Selection Process of Equine TMJ OA Biomarkers

Summarizing the main biomarkers of TMJ OA and considering their role in TMJ OA pathogenesis (Table 2), the following keywords for literature search were selected: ADAMTS-5 (ADAMTS), Col2, HIF-1, HMGB2, Ihh, IL-1β, IL-6, IL-12 (IL), MMP-3, MMP-13 (MMP), PGE2, PTHrP, TGF-β (TGF), TIMP-1 (TIMP), TNF-α (TNF), and VEGF. The keyword list was then extended by signaling pathway abbreviations: TGF-β/BMP (TGF, BMP), NF-κB, FGF, Wnt/β-Catenin (Wnt, β-Catenin), Ihh, and Notch. Duplicates (TGF, Ihh) were removed.

A literature search was performed using major information sources, including PubMed, Google Scholar, and ScienceDirect. The search queries involved biomarker *keyword* searches in combination with (temporomandibular* or TMJ*) and (horse* or equine*) and (arthritis* or osteoarthritis* or OA*), (degeneration* or disease*). Each *keyword* ((ADAMTS-5* or ADAMTS* or a disintegrin and metalloproteinase production with thrombospondin motifs*), (Col2* or collagen type II*), (HIF-1* or hypoxia-induced transcription factor*), (HMGB2* or high mobility group protein), (IL-1β* or IL* or interleukin*), (IL-6* or IL* or interleukin*), (IL-12* or IL* or interleukin*), (MMP-3* or MMP* or matrix metalloproteinase*), (MMP-13* or MMP* or matrix metalloproteinase*), (PGE_2_* or prostaglandin E_2_*), (PTHrP* or parathyroid hormone-related peptide*), (TIMP-1* or TIMP* or tissue matrix metalloproteinase inhibitors*), (TNF-α* or TNF* or tumor necrosis factor*), (VEGF* or vascular endothelial growth factor*), (TGF-β/BMP* or TGF-β* or TGF* or transforming growth factor* or BMP* or bone morphogenic protein*), NF-κB, FGF, (Wnt/β-Catenin* or Wnt* or β-Catenin*), Ihh, Notch) was combined separately. Due to the low number of gathered records, search queries involving combinations with (temporomandibular* or TMJ*) and (horse* or equine*) were performed. Additionally, the manual searches of reference lists from the included articles were performed.

A total of 52 articles were gathered from the period until 2023. Duplicates were removed. The inclusion criterion was the English language of articles. The exclusion criteria were article not related to horses, article not relevant to the aims of this review, published conference abstracts, and articles in journals that did not have a documented, transparent peer-review process. From the gathered articles, only four covered the review area of equine TMJ OA biomarkers.

### 3.2. Biomarkers of Equine TMJ OA

Most equine TMJ research focuses on joint morphology and function [10,38,39,40,41,57,58] and imaging modalities [6,7,41,61,62], as well as practical implications and treatment [34,36,37,59,63]. Only a few key biomarkers of equine TMJ OA [5,9,25,137] have been investigated and they are summarized in Table 3. While these studies effectively address the clinical needs of equine medicine, the similarity to the underlying molecular components of human TMJ OA is still poorly understood.

Carmalt et al. [5] investigated IL-1, IL-6, IL-8, TNF-α, and TGF-β in the synovial fluid collected from clinically healthy horses. The horses were grouped in two ways: based on age and based on dental diseases. This grouping was guided by the equine dentistry hypothesis, which mirrors concepts in human dentistry [138], suggesting that dental diseases may lead to clinically manifested TMJ OA, including pain and reduced performance. However, this study failed to confirm the hypothesis, as no dental-disease-related differences were demonstrated in any of the examined biomarkers. In this study, the concentrations of IL-8 and TGF-β1 increased with age. The authors speculated that this rise in IL-8 and TGF-β concentration could indicate low-grade inflammation associated with dental wearing, even in the absence of typical OA clinical symptoms. However, it is worth noting that not all TMJs underwent a detailed examination and they were not grouped based on OA symptoms. Therefore, establishing a direct relationship with TMJ OA may be considered controversial [5].

Cota et al. [9] assessed changes in the composition and mechanical properties of the articular disc in relation to location, age, and OA severity. This post mortem study aimed to characterize the articular disc to enhance the understanding of TMJ function and the potential role of articular disc degradation in equine TMJ OA. Among the evaluated indicators of biochemical composition, histological structure, and compressive properties, sulfated glycosaminoglycan (GAG) and total collagen (TC) may be considered as informative biomarkers, if not main ones. The authors observed regional variations in articular disc composition and compressive stiffness, with increasing GAG content related to increasing compressive stiffness. Thus, the authors speculated that, in horses, the caudal part of the TMJ may be more susceptible to degeneration. In this study, the articular disc exhibited increasing GAG content and compressive stiffness with increasing age, and a region-specific increase in GAG content associated with OA severity [9].

Carmalt et al. [25] expanded on their previous research [5] by employing a chemically induced OA model. The authors investigated IL-6, TNF-α, TGF-β, and total protein (TP) in the synovial fluid collected from the TMJ and the MCP joint, known to be frequently affected by OA. In this study, TNF-α concentration was higher, whereas TP concentration was lower, in the TMJ OA compared to the control TMJ. However, both biomarker concentrations were higher in the TMJ OA than in the MCP OA. In contrast, TGF-β concentration was lower in the TMJ OA than in the MCP OA. Thus, the authors suggested that the TMJ responds differently to acute OA than peripheral joints but they were unable to precisely explain the reason for the observed differences, suggesting that further research concerning MMPs and TLR-4 is required [25]. Therefore, we can support this suggestion by expanding research on the NF-κB signaling pathway, since this pathway is activated by TLR-4 and may regulate the transcription of MMPs, ILs, and TNF-α [109,113,129].

Pereira et al. [137] explored a load-induced OA model to mimic the TMJ stresses typical of routine equine dental care. Considering that overload may initiate degenerative changes in the TMJ [15,76], the authors hypothesized that the application of a full-mouth speculum for 60 min may lead to clinical symptoms of TMJ OA. Among the main biomarkers, the authors investigated PGE_2_ in the synovial fluid and, additionally, TP, GAG, chondroitin sulfate (CS), hyaluronic acid (HA), and white blood cells (WBC) were also assessed in the synovial fluid. In this study, only WBC count was higher after load induction. However, the lack of clinical symptoms and thermographic signs of OA suggests that the used load-time was too short for disease induction, and such a load remains in the range of TMJ adaptability. This observation is beneficial for horse owners and practitioners, ensuring the safety and lack of PGE_2_-mediated pain during dental care procedures. The authors concluded that the used load does not indicate the occurrence of turnover changes in articular cartilage; however, they suggested the need for monitoring each horse subjected to prolonged full-mouth speculum placement [137]. We can support this suggestion by using overload biomarkers, including HMGB2, HIF-1, VEGF, MMPs, and TIMP-1 [82,93,94,95], which may make it feasible to expand further research on the safety of routine and interventional equine dental care.

## 4. Limitations and Future Directions

Despite the discussed advantages of the equine TMJ OA model, the following disadvantages should be considered. Recently, challenges such as the difficulty and expense of housing and managing horses [139], as well as the high cost, long research period, and slow disease progression [14], have been highlighted. While it is acknowledged that horses represent an expensive experimental model, the numerous studies referenced focusing on both equine athletes and experimental horses suggest that they are manageable subjects [5,6,7,8,25,34,36,37,38,39,40,41,59,61,62,64,137].

However, none of the previous reviews of animal models have highlighted the functional differences between human and equine TMJ. Different mandibular movements occur during mastication in humans, rodents, carnivores, and herbivores [140], while horses represent typical herbivores. The most significant difference is the restriction in lateral movement, which is most strongly expressed in rodents [35], present in carnivores [141], and least expressed in herbivores, while horses are capable of both latero-ventral movements and medio-dorsal movements [70,71]. Moreover, equine masticatory forces are higher [70] than those in humans [142], and both differ depending on the kind of food intake [71,143]. Horses usually consume roughage such as grass and hay, along with concentrates, most often based on oats. Harder additions may include a piece of dry bread, a carrot, and a horse cookie [71], whereas the human diet, considered in chewing studies, is much more varied [143,144,145]. Despite these and other functional differences, animal studies serve as a source for new insights into human TMJ diseases or for targeting novel treatment strategies [140].

The relatively high prevalence of TMJ OA and the longer lifespan of horses, compared to other domestic animals, offer an opportunity to examine potential age- and OA-related changes in the TMJ, making it easier to form research groups of different ages rather than tracking the slow progression of the disease over time [9]. Furthermore, recent developments in equine medicine, including the availability of helical fan beam CT imaging of the equine head in a standing position [146,147], have significantly increased the suitability of horses as model animals for TMJ OA. Therefore, the equine naturally occurring model appears to be feasible for studying TMJ OA, including cartilage degradation and bone remodeling. With improved biomarker evaluation, TMJ OA can provide valuable evidence for studying the pathogenesis of TMJ OA at different ages.

One may observe that the value of animal models mainly depends on how well they correspond with human disease. Therefore, improving animal TMJ OA models may be considered the primary means of testing potential therapeutic agents to determine their potential efficacy in this specific disease [14,16]. On the other hand, evaluating treatments in clinical equine practice [6,7,34,36,37,40,59,64] faces similar challenges to those in humans [35]. In both cases, high-quality randomized, controlled trials with well-validated outcome measures are needed to improve the investigation of the efficacy of TMJ OA treatment strategies.

Currently, the treatments of TMJ OA mainly aim to reduce pain, restore TMJ function, and improve the quality of life of patients [1,15,19,67]. Given the limited understanding of TMJ OA pathogenesis and the limited regeneration possibility of the articular cartilage, no clinically approved therapeutics are still available to restore the TMJ structure [1,45,89]. Giving the example of a possible therapeutic target in the signaling pathway of TMJ OA, one may observe that an inhibition of TGF-β signaling may delay the effect of mechanical load on cartilage degradation in TMJ OA [148]. Inhibition of NF-κB signaling protects condylar cartilage from degradation [149] and ameliorates chondrocytes during TMJ inflammation [150]. The inhibition of FGF signaling in chondrocytes delays and ameliorates TMJ OA progression by promotion of the autophagic activity of chondrocytes [118]. The inhibition of Wnt/β-catenin signaling promotes cell proliferation and inhibits cell death in articular cartilage [122]. The inhibition of Ihh signaling prevents chondrocyte differentiation and maturation [151] and chondrocyte terminal differentiation in TMJ OA, delaying the overload OA lesions [124]. Finally, the inhibition of Notch signaling delays the progression of cartilage damage in TMJ OA [146].

However, all these studies were performed on mice [118,122,148,149,150,151,152] and rat [124,149] models, focusing on the identification of symptom-modifying OA drug (SMOAD) effects rather than the disease-modifying activity of the drug [35]. Thus, there is a need to develop pre-clinical animal models for research on a disease-modifying OA drug (DMOAD) that will better correspond with human TMJ OA progression and outcomes after treatment [14,19,35]. Such models should include long-term cartilage and bone structure recovery, biomechanical analyses concerning functional joint recovery [14], and validatable and detailed imaging modalities, as well as synovial fluid and serum biomarker evaluation [12,16,35] to define DMOAD activity. Moreover, the use of recently available multiplex panels rather than individual ELISAs and non-targeted transcriptomic techniques enable the expanded evaluation of OA biomarkers [153,154,155], so also TMJ OA biomarkers [156,157].

As equine sports medicine develops rapidly parallelly to human medicine, the equine TMJ OA model potentially offers high-quality research and well-validated outcome measures using, for example, dual-energy, multidetector standing fan-beam CT and high-field MRI. Therefore, every effort should be made to fill the gaps in the biomarkers and signaling pathways of equine research, and establish more multi-factor models of equine TMJ OA. So, the hope is that further studies on TMJ OA in horses provide great potential for targets of regenerative treatment for advancement in both human and equine medicine.

## 5. Conclusions

Different types of animal models simulate different pathological expressions of TMJ OA and have their unique characteristics. Currently, mice, rats, and rabbits are commonly used in the study of TMJ OA; however, naturally occurring large-animal models are still needed for preclinical studies to evaluate the clinical processes and treatment of TMJ OA. Among the naturally occurring models, equine TMJ OA is characterized by spontaneous OA occurring; morphological and functional similarities to human TMJ structures; and a wide range of clinical examinations and imaging modalities that can be performed on horses. However, the numerous biomarkers of disease progression in equine TMJ OA model require further research. Among the main TMJ OA biomarkers, IL-1, IL-6, TGF-β, TNF-α, and PGE_2_ have recently been investigated in the equine model. However, the majority of biomarkers for cartilage degradation (MMPs, TIMP-1, ADAMTSs), chondrocyte apoptosis (TNF-α) and hypertrophy (Col2, Ihh, PTHrP), angiogenesis (VEGF), and TMJ overload (HMGB2, HIF-1, VEGF, MMPs, TIMP-1), as well as the main signaling pathways (TGF-β/BMP, NF-κB, FGF, Wnt/β-Catenin, Ihh, and Notch), have not been studied so far. Considering the disadvantages of equine TMJ OA, mainly related to high costs and long disease progression, it would be advisable to focus further research on horse specimens, considering HMGB2, HIF-1, VEGF, MMPs, TIMP-1, and ADAMTSs, as well as NF-kB and Ihh signaling pathways. Although TMJ OA is underinvestigated in equine medicine, it has great potential in targeted treatment research.

## Figures and Tables

**Table 1:** Summary of the six main signaling pathways in temporomandibular osteoarthritis (TMJ OA).

Signaling	Members	Biological Role	Activation	Role in TMJ OA
TGF-β/BMP	Over forty members: TGF-βs, BMPs, activin [104]	Modulation of bone or cartilage production and modeling [105]	Activation of TGF-β /Smad3 signaling [106]; degradation of Col2 [86]; increase secretion of TGF-β [107]	Cartilage degradation [106,108]; chondrocyte hypertrophy [86]; subchondral bone sclerosis [105,107]
NF-κB	RelA, RelB, c-Rel, NF-κB1, NF-κB2 [109]; engaging TNF-R, TLR, TCL [109], and RANKL [110]	Mediation of inflammatory responses, cell proliferation, and cell death [109]	Increase transcription of MMPs, cytokines [111], and ADAMTS-5 [89]; increase transcription of osteopontin, stimulate MMP production [112]; increase transcription of IL-1β and IL-6, stimulate VEGF secretion [102]; increase transcription of IL-1β, stimulate MCP-1 secretion [100]; increasing RANKL modulate osteoclast production by TNF-α, IL-1β, and IL-17 secretion [97]	Cartilage degeneration [89,112,113]; subchondral bone sclerosis [97,113]; inflammation [100,102,111], angiogenesis [102]
FGF	FGFs [114]; engaging PI3K, PLC, STAT, MAPK [115]	Regulation of skeletal development [114], predominately articular cartilage [116]	Activation of death receptor (MEK/ERK) pathway [117]; increase transcription of TNF-α [117,118]	Chondrocyte apoptosis [118]
Wnt/β-Catenin	Wnt glycoprotein, β-Catenin, LRP5/6 [119]	Regulation of cell proliferation and differentiation [120]	Increase transcription of MMP-13, ADAMTS-4, and ADAMTS-5 [12,121]	Cartilage degeneration [12,121]; chondrocyte apoptosis [122]
Ihh	Hh proteins [123,124]	Regulation of skeletal development [125], predominately chondrocyte in cartilage [126]	Increase transcription of Ihh and PTHrP [124]	Chondrocyte hypertrophy [27,124] induced by the mechanical load on cartilage [124]
Notch	Notch ligands, Notch receptors, and transcriptional effectors [127,128]	Regulation of cell differentiation and apoptosis [127]; involved in cartilage synthesis and degradation [129]	Increase transcription of MMP-13, IL-1β, and IL-6 [130]	Cartilage degeneration [131]; inflammation [132]; angiogenesis [130]

Abbreviations: ADAMTS, a disintegrin and metalloproteinase production with thrombospondin motifs; BMP, bone morphogenic protein; Col2, collagen type II; FGF, fibroblast growth factor; Hh, Hedgehog; Ihh, Indian hedgehog; IL, interleukin; LRP, low-density lipoprotein receptor-related proteins; MMP, matrix metalloproteinase; MAPK, mitogen-activated protein kinase; MCP-1, monocyte chemoattractant protein-1; NF-κB, nuclear factor kappaB; PTHrP, parathyroid hormone-related peptide; PI3K, phosphoinositide 3-kinase; PLC, phospholipase C; RANKL, receptor activator of nuclear factor kappa-B ligand; STAT, signal transducers and activators of transcription; TGF-β, transforming growth factor β; TLR, toll-like receptor; TNF-α, tumor necrosis factor α; TNF-R, TNF receptor.

**Table 2:** Summary of main biomarkers in temporomandibular osteoarthritis (TMJ OA).

Role in TMJ OA	Biomarkers
Cartilage degradation	MMP-3 [98,112,133,134,135], MMP-13 [12,27,98,112,118,121,130,133,135], TIMP-1 [82,94,95], ADAMTS-5 [2,12,27,89,118,121,133]; * (MMP-3, MMP-13 upregulated by IL-1β [98] and VEGF [94,95]), * (TIMP-1 downregulated by VEGF [94,95]), * (ADAMTS-5 upregulated by IL-6 [2])
Chondrocyte apoptosis	TNF-α [117,118,136]
Chondrocyte hypertrophy	Col2 [86], Ihh and PTHrP [124]
Subchondral bone sclerosis	TNF-α, IL-1β, and IL-17 [97], TGF-β [107]
Inflammation	IL-1β [96,97,98,99,100,101,102,130], IL-6 [2,102,130], IL-12 [96], TNF-α [97,98,99], MCP-1 [100,101] * (MCP-1 upregulated by IL-1β [100])
Angiogenesis	VEGF [82,83,94,102] * (VEGF upregulated by IL-1β, IL-6 [102], and HIF-1 [94])
Pain	IL-1β, TNF-α [99], PGE_2_ [103]
Overload	HMGB2 [93], sequence of HIF-1, VEGF, MMP-13, and TIMP-1 [82,94,95]

Abbreviations: ADAMTS-5, a disintegrin and metalloproteinase production with thrombospondin motifs 5; Col2, collagen type II; HIF-1, hypoxia-induced transcription factor-1; HMGB2, high mobility group protein B2; Ihh, Indian hedgehog; IL, interleukin; MMP, matrix metalloproteinase; MCP-1, monocyte chemoattractant protein-1; PTHrP, parathyroid hormone-related peptide; PGE_2_, prostaglandin E_2_; TIMP-1, tissue matrix metalloproteinase inhibitors; TGF-β, transforming growth factor β; TNF-α, tumor necrosis factor α; VEGF, vascular endothelial growth factor. * Upregulation or downregulation by other listed biomarkers.

**Table 3:** Summary of biomarkers investigated in temporomandibular osteoarthritis (TMJ OA) in equine specimens.

Biomarkers	TMJ OA Model	Demographics	Methods	Results	Reference
IL-l, IL-6, IL-8, TNF-α, TGF-β	No OA-related grouping; Age-related grouping; Dental disease-related grouping	25 horses 0.25 to 21 years	Synovial fluid; LM-l cell proliferation assay (IL-1), TTDI cell proliferation assay (IL-6), microchemotaxis assay (IL-8), L929 cell cytotoxicity assay (TNF-α), ELISA (TGF-β)	IL-8 and TGF-β concentration increased with horses’ age.	[5]
GAG, TC	Naturally occurring OA; OA severity-related grouping	16 horses 5 to 25 years	Intra-articular disc (fibrocartilaginous); DMMB assay (GAG), hydroxyproline assay (TC)	GAG content was higher in severe OA than in normal TMJ or mild OA. GAG content increased with horses’ age.	[9]
IL-6, TNF-α, TGF-β, TP	Chemically induced OA model in the TMJ and the MCP	7 horses 5 to 10 years	Synovial fluid; ELISA	TNF-α concentration was higher in the TMJ OA than control TMJ. TP concentration was lower in the TMJ OA than control TMJ. TNF-α and TP concentrations were higher in the TMJ OA than the MCP OA. TGF-β concentration was lower in the TMJ OA than the MCP OA.	[25]
PGE_2_, WBC, TP, GAG, HA, CS	Mechanical loading OA (1 h open month load)	12 horses 12.1 ± 1.5 years	Synovial fluid; Neubauer chamber (WBC), Bradford method (TP), EIA (PGE_2_), DMMB assay (GAG, HA, CS)	Only WBC was higher 5 h after 1 h open month load.	[137]

Abbreviations: CS, chondroitin sulfate; DMMB, 1,9-dimethylmethylene blue binding assay; ELISA, enzyme-linked immunoassays; EIA, enzyme-linked immunosorbent assay; HA, hyaluronic acid; IL-l, interleukin-1; IL-6, interleukin-6; IL-8, interleukin-8; MCP, metacarpophalangeal joint; OA, osteoarthritis; PGE_2_, prostaglandin E_2_; GAG, sulfated glycosaminoglycan; TMJ, temporomandibular joint; TP, total protein; TC, total collagen; TGF-β, transforming growth factor β; TNF-α, tumor necrosis factor α; WBC, white blood cell.

## Data Availability

Not applicable.
